# β-Nicotinamide mononucleotide protects against hypervirulent *Klebsiella pneumoniae* bloodstream infection and liver injury

**DOI:** 10.1128/msphere.00361-25

**Published:** 2025-07-31

**Authors:** Zubai Cao, Bao Meng, Jie Chen, Ruomu Ge, Yanyan Liu, Jiabin Li

**Affiliations:** 1 Department of Infectious Diseases & Anhui Center for Surveillance of Bacterial Resistance, The First Affiliated Hospital of Anhui Medical University 36639, Hefei, China; 2 Anhui Province Key Laboratory of Infectious Diseases & Institute of Bacterial Resistance, Anhui Medical University 12485, Hefei, China; University of Nebraska Medical Center College of Medicine, Omaha, Nebraska, USA

**Keywords:** hypervirulent *Klebsiella pneumoniae*, bloodstream infection, NMN, liver abscess

## Abstract

In clinical practice, hypervirulent *Klebsiella pneumoniae* (hvKP) often causes bloodstream infections (BSI) and liver abscesses; however, the mechanism of hvKP-induced liver abscess formation remains unclear. β-Nicotinamide mononucleotide (NMN) has been reported to have potential therapeutic effects on sepsis, but its impact on hvKP BSI is still unclear. Supplementation with NMN improved the survival rate of hvKP BSI, and the protective effects of NMN disappeared after macrophage depletion. NMN treatment can reduce NF-κB signaling pathway activation in bone marrow-derived macrophages (BMDM) after hvKP infection, as well as the transcription of *Il6*, *Il1b*, *Tnf*, and *Cxcl2*. NMN, combined with antibiotic therapy, blocked the formation of hvKP liver abscesses. The treatment regimen of NMN combined with antibiotics effectively reduced the levels of inflammatory factors in the liver, inhibited the activation of the NF-κB signaling pathway, and reduced the infiltration of neutrophils into the liver. These results indicate that NMN exerts a protective effect against hvKP BSI by inhibiting excessive inflammatory response, and NMN has therapeutic potential for preventing liver injury caused by hvKP.

**IMPORTANCE:**

This study found that β-nicotinamide mononucleotide (NMN) can protect mice against hvKP BSI via inhibiting the NF-κB signaling pathway activation. Supplementing NMN with antibiotic treatment alleviated the inflammatory response of the liver and reduced the formation of liver abscess, providing insight into the mechanism of liver abscess research.

## INTRODUCTION

Bloodstream infections (BSI) are one of the main causes of sepsis and septic shock and remain a challenge in clinical treatment (1). *Klebsiella pneumoniae* (KP) is a commonly detected strain in BSI. Patients infected with hypervirulent *Klebsiella pneumoniae* (hvKP) are often admitted to the hospital due to liver and intraocular abscesses or central nervous system infections, with liver abscesses being the most common. The occurrence of an abscess leads to increased pain in and prolonged hospital stay for patients (2); therefore, it is necessary to study the formation mechanism of hvKP liver abscesses.

Macrophages are the most important line of defense against hvKP BSI. Xueting Huang et al. and Chu Han Hoh et al. demonstrated the clearance effect of macrophages on hvKP using macrophage-cleared liposomes (3, 4). However, the role and mechanisms of macrophages in liver injury and abscess formation are still unknown.

Wanford et al*.* used fluorescence microscopy and *ex-vivo* pig organ model and found that hvKP resisted Kupffer cell-mediated clearance and triggered recruitment of neutrophils, providing research ideas and entry points for animal studies of liver abscesses (5). Hullahalli et al. directed their research toward macrophages and neutrophils using spatial transcriptomic and single-cell RNA sequencing in a liver abscess model induced by *Escherichia coli* (6). They defined the *E. coli* abscess formation as the simultaneous presence of visible white lesions and a hepatic bacterial load ≥ 10⁴ colony-forming units (CFU) in their study, and hematoxylin and eosin (HE) staining displayed that the abscess core was predominantly composed of necrotic hepatocytes encircled by a mixed inflammatory cells infiltrate, including macrophage- and neutrophil-like cells. They also found that TLR4 knockout mice were resistant to *E. coli* liver abscess formation, suggesting that the formation of liver abscesses is likely related to excessive activation of the innate immune response.

Nicotinamide adenine dinucleotide (NAD^+^) is an important coenzyme for redox reactions. β-Nicotinamide mononucleotide (NMN) is a direct precursor of NAD^+^ in the NAD^+^ salvage pathway *in vivo*, which can effectively supplement NAD^+^ levels, and the levels of NMN are closely associated with nicotinamide phosphoribosyltransferase (NAMPT) activity *in vivo* (7). NMN can exert anti-inflammatory and anti-oxidant effects in various disease models, including sepsis models, where NMN can reduce systemic inflammatory cytokine levels and oxidative damage, thereby protecting organ function (8). We speculated whether NMN may reduce mortality from hvKP infection and reduce liver injury by alleviating inflammatory responses. In this study, we showed that NMN can improve the survival rate of hvKP BSI mice and evaluated the protective effect of NMN on hvKP BSI liver injury using an antibiotic combination therapy model.

## RESULTS

### Supplementing NMN to protect mice from hvKP BSI

**Fig 1:**
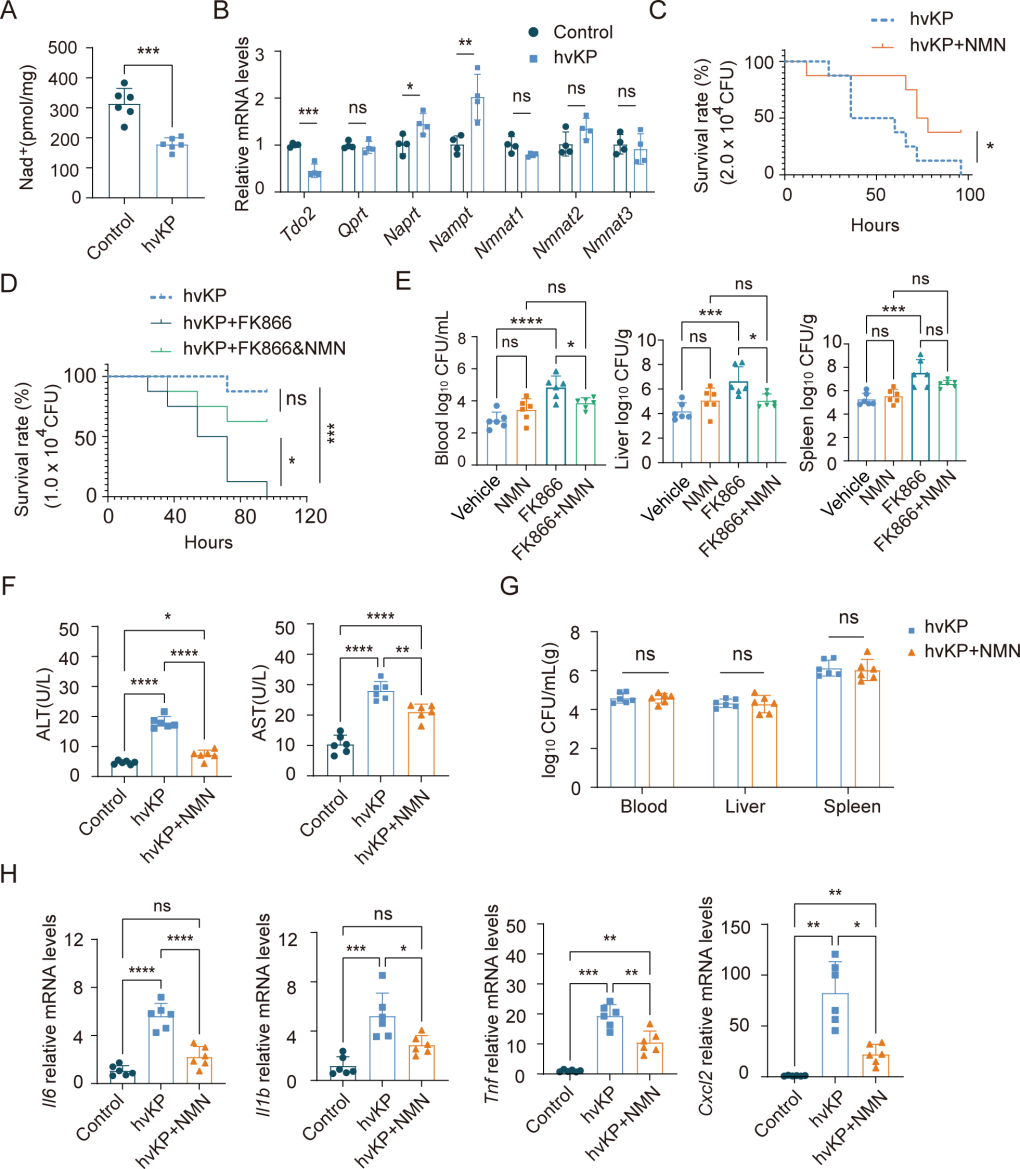
Supplementation of NMN to protect mice from hvKP BSI. Tail vein injection with 1.0 × 10^4^ CFU of ATCC 43816. NMN (185 mg/kg, i.p.) was supplemented 1 h before infection, or NAMPT inhibitor FK866 (30 mg/kg, i.p.) was administered 3 h after infection. (**A and B**) NAD^+^ (*n* = 6) and mRNA levels of *Tdo2*, *Qprt*, *Naprt*, *Nampt*, *Nmnat1*, *Nmnat2*, and *Nmnat3* in liver at 24 hpi (*n* = 4). (**C**) Survival rates of NMN-supplemented group and untreated group infected with 2.0 × 10^4^ CFU of ATCC 43816 (*n* = 8). (**D and E**) Survival rate of hvKP BSI in different groups (*n* = 8) and bacterial load in the circulation and visceral organs in different groups at 24 hpi (*n* = 6). (**F and G**) Levels of ALT, AST, and bacterial load in the circulation and visceral organs between NMN-supplemented group and untreated group at 6 hpi (*n* = 6). (**H**) mRNA levels of *Il6*, *Il1b*, *Tnf*, and *Cxcl2* in liver at 4 hpi (*n* = 6). Data are representative results from three independent experiments (mean  ±  SEM). *, *P* <  0.05; **, *P* <  0.01; ***, *P* <  0.001; ****, *P* < 0.0001; ns, not significant. NMN, β-nicotinamide mononucleotide; hvKP, hypervirulent *Klebsiella pneumoniae*; BSI, bloodstream infection; CFU, colony-forming units; hpi, hours post-infection. Control, no treatment or infection group; Vehicle, vehicle treatment and infection group.

Studies have shown that NAD^+^ levels are decreased in multiple organs in sepsis models. We found that hvKP infection decreased NAD^+^ in the mouse liver at 24 h post-infection (hpi) (Fig. 1A). Real-time reverse transcriptase-polymerase chain reaction (RT-qPCR) was performed on mouse livers to detect the mRNA levels of enzymes related to NAD^+^ synthesis. The results showed a decrease in the transcript level of *Tdo2* associated with *de novo* NAD^+^ biosynthesis and an increase in the transcript level of *Nampt* associated with the NAD^+^ salvage pathway (Fig. 1B). NAMPT catalyzes NMN synthesis from nicotinamide (NAM). To evaluate the role of NMN in hvKP BSI, we administered NMN via intraperitoneal injection 1 h before infection. The results showed that supplementation with NMN increased the survival rate of the mice (Fig. 1C). Furthermore, we treated mice with the intracellular NAMPT inhibitor FK866 to inhibit NMN biosynthesis. The results showed that FK866 increased the mortality rate of mice, which could be corrected by supplementation with NMN. This indicates that the NAMPT-NMN axis is necessary for mice to combat hvKP BSI (Fig. 1D). In terms of bacterial load, we found that supplementation with NMN did not significantly reduce colony-forming units (CFU) in mouse blood, liver, and spleen, but FK866 increased CFU in mouse blood and liver; supplementation with NMN before FK866 treatment reduced CFU in the blood and liver (Fig. 1E). We observed an increase in alanine aminotransferase (ALT) and aspartate aminotransferase (AST) levels in the sera of mice 6 h after hvKP infection, whereas treatment with NMN reduced these levels (Fig. 1F). However, NMN did not reduce the CFU levels in the blood, liver, or spleen at 6 hpi (Fig. 1G). We detected liver inflammatory cytokine mRNA levels and found that NMN supplementation significantly reduced expression of *Il6*, *Il1b*, *Tnf*, and *Cxcl2* at 4 hpi (Fig. 1H). These results indicated that hvKP infection leads to a decrease in NAD^+^ levels in the livers of mice and activates the NAD^+^ salvage pathway. Supplementation with NMN can protect mice from hvKP BSI and alleviate liver inflammation and injury, whereas inhibition of NMN biosynthesis can exacerbate mouse mortality.

### The protective effect of NMN on the survival rate of hvKP BSI depends on macrophages

**Fig 2:**
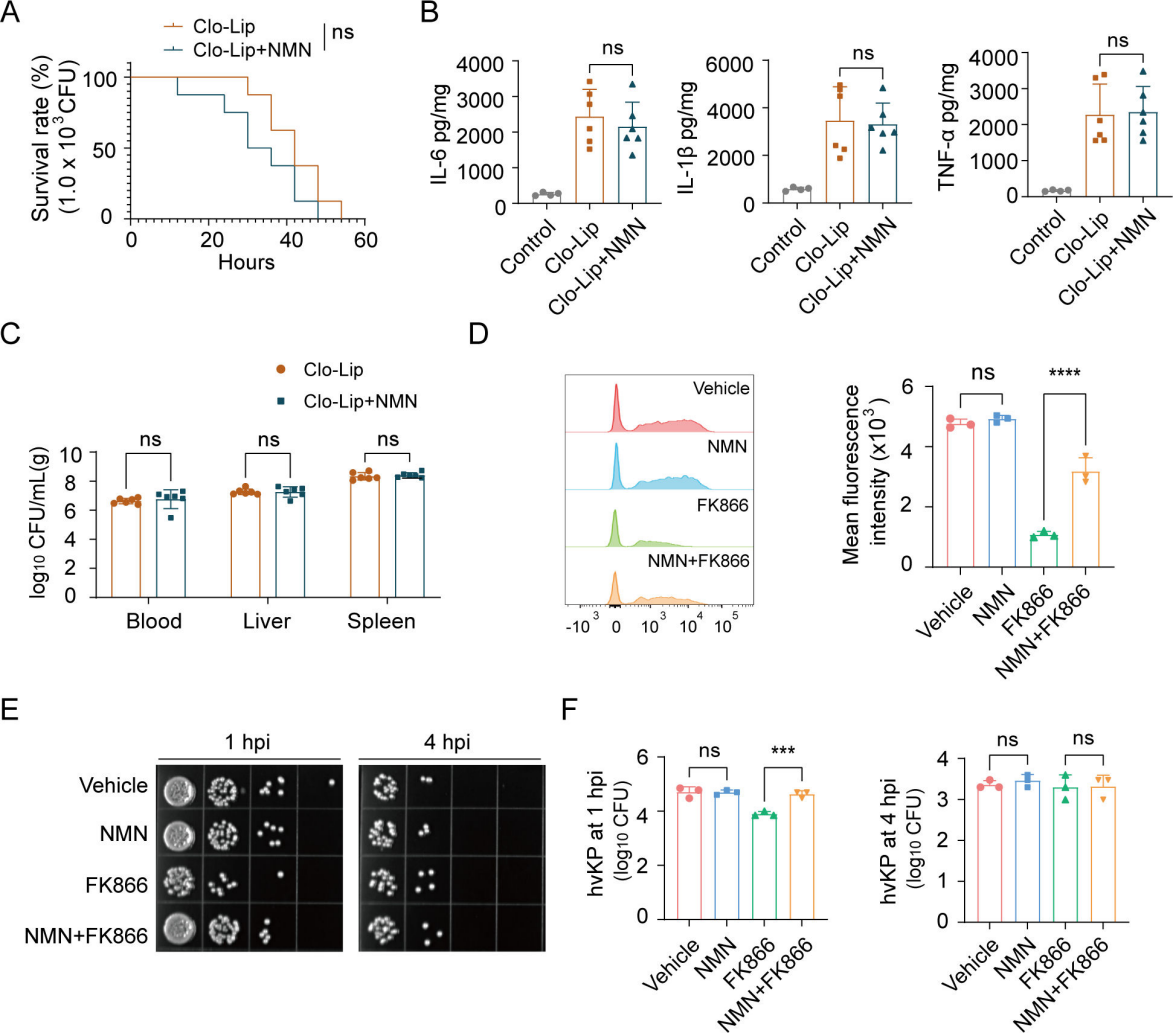
The protective effect of NMN on the survival rate of hvKP BSI depends on macrophages. (**A**) Survival rate of hvKP BSI in macrophage-depleted mice with or without NMN treatment (*n* = 8) (1.0  ×  10^3^ CFU). (**B and C**) Levels of IL-6, IL-1β, and TNF-α and bacterial load in the circulation and visceral organs in macrophage-depleted mouse liver with or without NMN treatment at 24 hpi (*n* = 6). (**D**) Phagocytic capacity of marrow-derived macrophages (BMDMs) from NMN (500 µM)- or FK866 (25 nM)-treated and vehicle controls using flow cytometry analysis at 1 hpi. (**E and F**) Gentamicin protection assay, intracellular bacterial counts of BMDMs from NMN (500 µM)- or FK866 (25 nM)-treated and vehicle controls at 1 and 4 hpi. Data are representative results from three independent experiments (mean  ±  SEM). *, *P* <  0.05; **, *P* <  0.01; ***, *P* <  0.001; ****, *P* < 0.0001; ns, not significant. NMN, β-nicotinamide mononucleotide; hvKP, hypervirulent *Klebsiella pneumoniae*; BMDM, bone marrow-derived macrophage; BSI, bloodstream infection; Clo-Lip, clodronate liposomes; CFU, colony-forming units; hpi, hours post-infection; MOI, multiplicity of infection; Control, no treatment or infection group; Vehicle, vehicle treatment and infection group.

To observe whether the protective effect of NMN is achieved through macrophages, we found that after pre-clearing macrophages with clodronate liposomes (Clo-Lip), NMN could no longer protect the survival rate (Fig. 2A). After macrophage depletion, there was no difference in the levels of IL-6, IL-1β, and TNF-α in mouse liver between the two groups, and supplementation with NMN did not alter the bacterial load in the blood, liver, and spleen (Fig. 2B and C), indicating that the protective effect of NMN on hvKP BSI depends on macrophages.

To further evaluate the effect of NMN on macrophage bacterial clearance, we treated bone marrow-derived macrophages (BMDM) with NMN and FK866, used GFP-labeled hvKP, and evaluated the phagocytic ability of macrophages using flow cytometry at 1 hpi. The results showed that NMN pretreatment did not increase the phagocytic abilities of BMDM toward hvKP; FK866 pretreatment weakened the phagocytic abilities of macrophages, which could be partially offset by supplementation with NMN (Fig. 2D). The results of the gentamicin protective assay were consistent with this finding; FK866 treatment significantly reduced intracellular hvKP CFU counts at 1 hpi, and the effect was reversed by NMN supplementation. Compared to the 1 hpi, we observed an approximately one log10 reduction in intracellular bacterial counts in all groups, except for the FK866-treated group, at 4 hpi (following gentamicin-mediated clearance of extracellular hvKP). Nonetheless, all treatment groups showed comparable intracellular CFU counts at 4 hpi, and we did not observe an increase in CFUs in the FK866 treatment group (Fig. 2E and F). These results indicated that the NAD^+^ salvage pathway is involved in the macrophage response to hvKP infection.

### NMN reduces the inflammatory response of macrophages infected with hvKP

**Fig 3:**
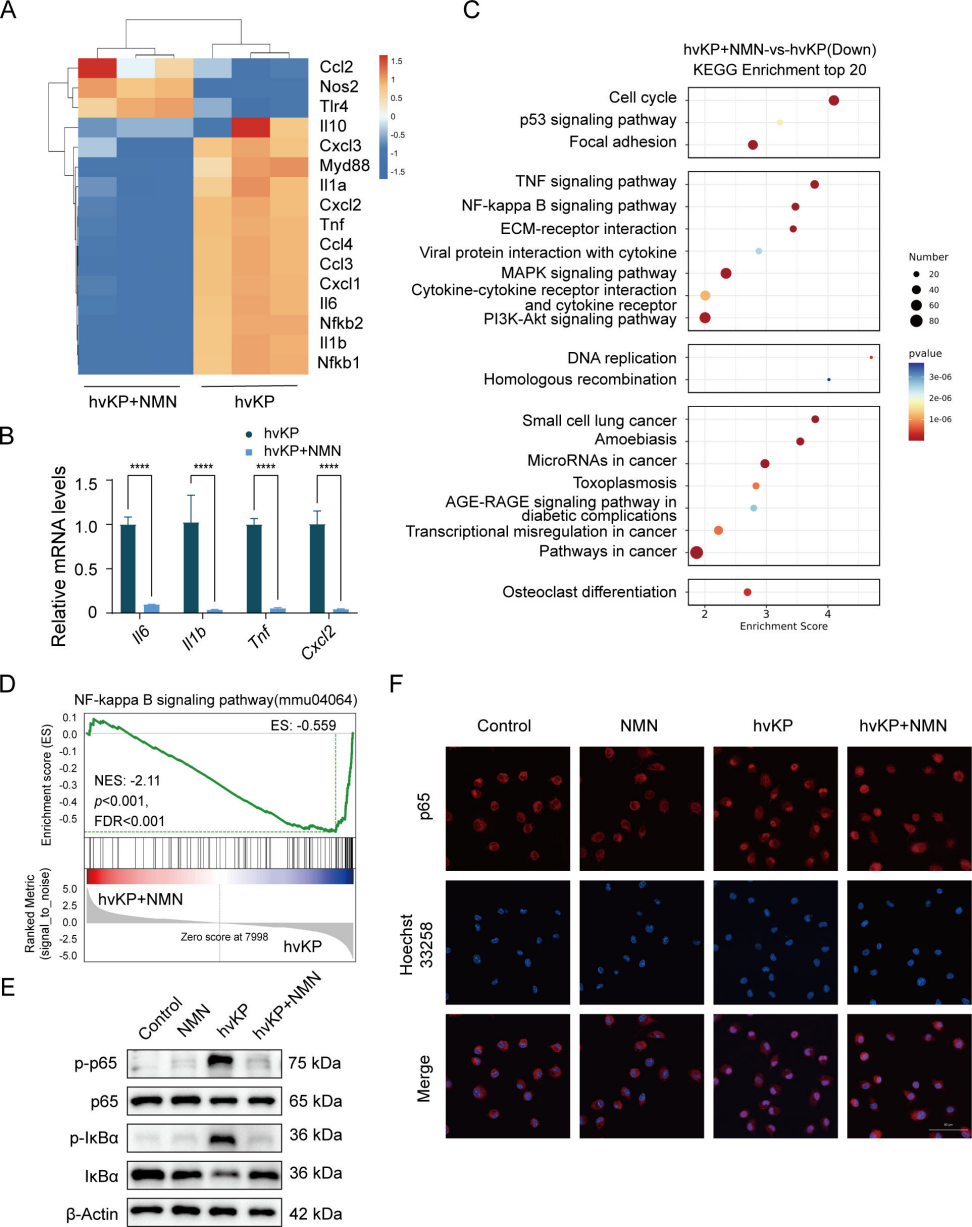
NMN reduces the inflammatory response of macrophages infected with hvKP. Marrow-derived macrophages (BMDMs) were treated with NMN (500 µM) for 16 h and infected with ATCC 43816 for 3 h (MOI = 1). (**A**) Hierarchical clustering heatmap of selected significant differentially expressed genes (DEGs) in BMDMs from NMN-treated and untreated controls. Red denotes increased expression; blue denotes decreased expression. (**B**) mRNA levels of *Il6*, *Il1b*, *Tnf*, and *Cxcl2* between NMN-treated and untreated controls. (**C**) Downregulated KEGG pathway enrichment after treatment with NMN. (**D**) GSEA analysis of NF-κB signaling pathway. (**E**) Western blot analysis of p-p65, p65, p-IκBα, and IκBα protein expression levels among four groups. (**F**) Immunofluorescence analysis of colocalization between p65 and nucleus (Hoechst 33258). Data are representative results from three independent experiments (mean  ±  SEM). *, *P* <  0.05; **, *P* <  0.01; ***, *P* <  0.001; ****, *P* < 0.0001; ns, not significant. NMN, β-nicotinamide mononucleotide; BMDM, bone marrow-derived macrophage; hvKP, hypervirulent *Klebsiella pneumoniae*; MOI, multiplicity of infection; Control, no treatment or infection group.

Next, we compared the genome-wide transcriptional sequencing results of BMDM infected with hvKP after NMN pretreatment. Pretreatment with NMN reduced the mRNA levels of *Myd88*, *Il6*, *Il1b*, *Tnf*, *nfkb1*, and *nfkb2* (Fig. 3A). Using RT-qPCR, we found that the mRNA levels of inflammatory factors related to neutrophil recruitment, such as *Il6*, *Il1b*, *Tnf*, and *Cxcl2*, were decreased after NMN treatment (Fig. 3B). The Kyoto Encyclopedia of Genes and Genomes (KEGG) pathway analysis showed that pretreatment with NMN downregulated the NF-κB and TNF signaling pathways, cytokine-cytokine receptor interaction, MAPK signaling pathway, and other signaling pathways closely related to bacterial infection in BMDM after hvKP infection (Fig. 3C). The gene set enrichment (GSEA) analysis suggests the downregulation of the NF-κB signaling pathway after NMN treatment (Fig. 3D); thus, we examined NF-κB signaling pathway activation in BMDMs. The western blot‌ (WB) results indicate that NMN pretreatment can significantly reduce the phosphorylation of p65 and IκBα in BMDMs (Fig. 3E). Simultaneously, we observed the intracellular localization of p65 in the nucleus through immunofluorescence and found that NMN pretreatment reduced the nuclear import of p65 (Fig. 3F). These results indicated that NMN pretreatment inhibited NF-κB pathway activation and inflammatory factor production.

### Supplementing NMN combined with antibiotic treatment to alleviate liver injury and abscess caused by hvKP

**Fig 4:**
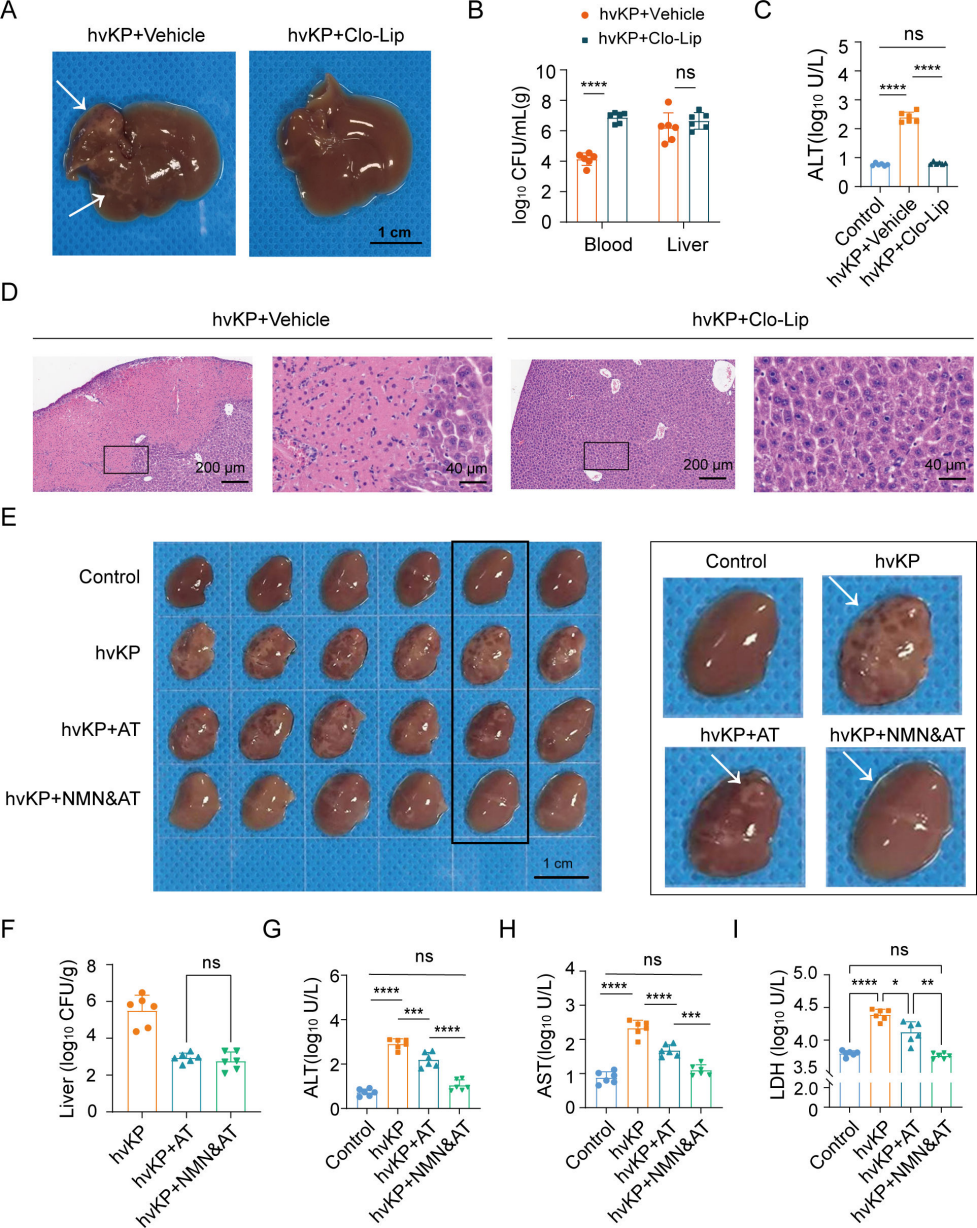
Supplementation of NMN combined with antibiotic treatment to alleviate liver injury and abscess caused by hvKP. (**A through D**) Liver, bacterial load, ALT levels, and HE staining of liver tissue in mice infected with 1.0 × 10^3^ CFU of ATCC 43816 at 18 hpi with or without clodronate liposome treatment (*n* = 6). (**E**) NMN (500 mg/kg, i.p.) was supplemented 1 h before infection and gentamicin (50 mg/kg, i.p.) was administered at 4.5 hpi, and mice were euthanized at 24 hpi. Mouse liver right upper lobes in different groups (*n* = 6). (**F**) Bacterial load of liver tissue in different groups (*n* = 6). (**G–I**) ALT, AST, and LDH levels in different groups (*n* = 6). Data are representative results from three independent experiments (mean ± SEM). *, *P* <  0.05; **, *P* <  0.01; ***, *P* <  0.001; ****, *P* < 0.0001; ns, not significant. NMN, β-nicotinamide mononucleotide; hvKP, hypervirulent *Klebsiella pneumoniae*; ALT, alanine aminotransferase; AST, aspartate aminotransferase; LDH, lactate dehydrogenase; AT, antibiotic treatment after hvKP infection; CFU, colony-forming units; hpi, hours post-infection. Control, no treatment or infection group; Vehicle, vehicle treatment and infection group.

We found that mice treated with clodronate liposomes (Clo-Lip) no longer developed irregular-shaped visible white lesions after hvKP infection (Fig. 4A). Clo-Lip increased the bacterial load in the mouse blood (Fig. 4B); however, ALT levels did not increase (Fig. 4C), and HE staining no longer showed areas of liver necrosis (Fig. 4D). This histological alteration observed in HE staining is consistent with previous characterizations of liver abscess studies, and we define the appearance of visible white lesions as the basic standard for abscesses in this study. Meanwhile, based on the above results, we speculate that the formation of hvKP liver abscess is related not only to bacterial load but also to macrophages and inflammation. We designed an antibiotic treatment (AT) model, and mice received intravenous injections with 1.5 × 10^4^ CFU of ATCC 43816 and administered 50 mg/kg gentamicin treatment at different time points. We found that antibiotic treatment within 3 hpi can block the formation of liver abscess. Antibiotic treatment at 4.5 hpi can completely eliminate bacteria from the blood at 24 hpi but cannot prevent abscess formation. Treating mice with NMN alone also cannot block the formation of liver abscess (Fig. S1A). Compared with the simple use of antibiotics, treatment with NMN combined with the administration of antibiotics significantly blocked the formation of hvKP liver abscesses (Fig. 4E). Antibiotic treatment effectively reduced liver bacterial load, but NMN did not further reduce it (Fig. 4F). In addition to gentamicin, we also attempted to use NMN in combination with colistin and meropenem, which also demonstrated a blocking effect on abscess formation, indicating that this effect is not specific to one particular antibiotic (Fig. S1B through E). In the *in vitro* checkerboard assay, NMN did not exhibit synergistic bactericidal effects with gentamicin, colistin, or meropenem, even at a concentration of 800 µg/mL (Fig. S1F). These indicated that NMN did not alleviate liver injury by enhancing bacterial clearance. We continued to detect biochemical indicators related to liver injury at 24 hpi, and the results showed that the combination of NMN and antibiotics reduced the levels of ALT, AST, and lactate dehydrogenase (LDH) significantly better than those of treatment with antibiotics alone (Fig. 4G–I). Next, we chose nicotinamide riboside (NR) and NAM, which are also considered NAD^+^ supplements, and combined them with antibiotics. NR and NAM successfully blocked the formation of hvKP liver abscesses in combination with antibiotics (Fig. S2A and B). We also evaluated whether supplementation with NR and NAM could improve the survival rate of mice with hvKP BSI and found that NR, but not NAM, could improve mouse survival (Fig. S2C). Considering that NR is converted to NMN *in vivo*, we will continue to conduct further studies on NMN.

### Supplementation of NMN combined with antibiotics effectively reduces liver neutrophil infiltration

**Fig 5:**
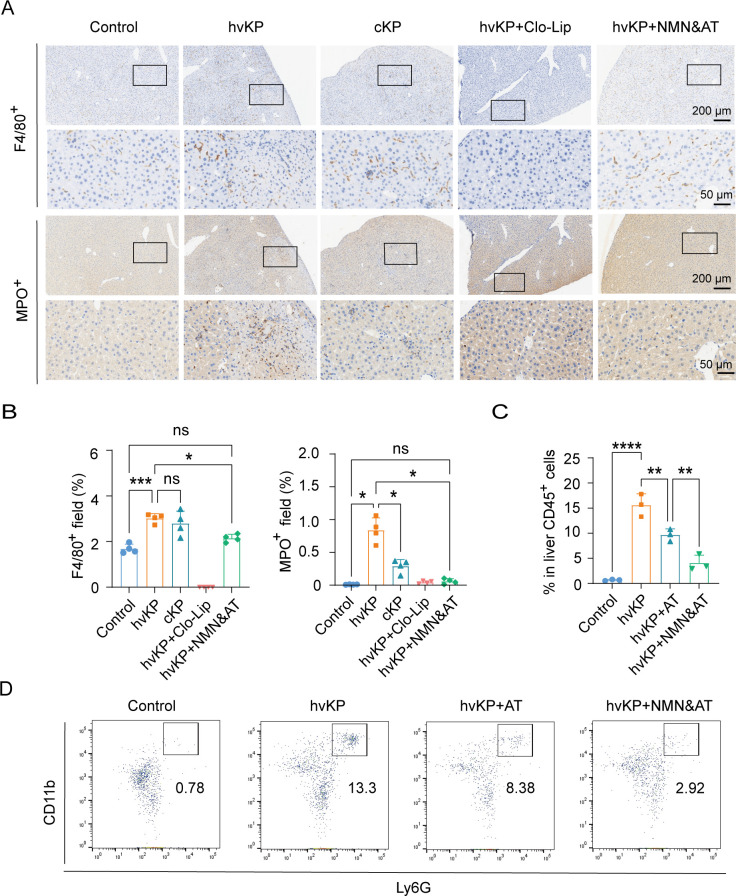
Supplementation of NMN combined with antibiotics effectively reduces liver neutrophil infiltration. (**A and B**) Immunohistochemical results of F4/80^+^ and MPO^+^ cells in liver in different groups (*n* = 4). (**C and D**) Proportion of CD11b^+^Ly6G^+^ cells to CD45^+^ cells in non-parenchymal liver cells via flow cytometry analysis (*n* = 3). Data are representative results from three independent experiments (mean  ±  SEM). *, *P* <  0.05; **, *P* <  0.01; ***, *P* <  0.001; ****, *P* < 0.0001; ns, not significant. NMN, β-nicotinamide mononucleotide; hvKP, hypervirulent *Klebsiella pneumoniae*; cKP, classical *Klebsiella pneumoniae*; Clo-Lip, clodronate liposomes; AT, antibiotic treatment after hvKP infection; Control, no treatment or infection group.

Considering that macrophages and neutrophils are the two most important immune cells involved in the formation of liver abscesses, we compared the distribution of F4/80^+^ and MPO^+^ cells between the healthy control group, hvKP infection group, classical KP (cKP) infection group (low-virulence), hvKP infection group after macrophage depletion, and NMN + antibiotics group using immunohistochemistry (Fig. 5A and B). Among these groups, only the hvKP group showed liver abscesses. Compared to the number of F4/80^+^ cells, the number of MPO^+^ cells in the cKP infection group was significantly lower than that in the hvKP infection group at 24 hpi. The results showed that there was significant infiltration of MPO^+^ cells in the necrotic area of the hvKP-infected group, and MPO^+^ cells were distributed in the hepatic sinusoids at high density, indicating a significant increase in the recruitment of MPO^+^ cells to the liver, which was not observed in other groups. This also aligns with the conventional definition of an abscess, and the combination of NMN and antibiotics significantly reduced MPO^+^ cell infiltration. To further evaluate the efficacy of NMN combined with antibiotics versus antibiotics alone, we used flow cytometry to compare the proportions of CD45^+^CD11b^+^Ly6G^+^ cells in liver non-parenchymal cells. The results also showed that the treatment regimen of NMN combined with antibiotics significantly reduced the proportion of CD11b^+^Ly6G^+^ cells among CD45^+^ cells (Fig. 5C and D). These results indicated that excessive recruitment of neutrophils is a significant feature of hvKP infection, and the combination of NMN and antibiotics can significantly reduce the recruitment of liver neutrophils caused by hvKP infection.

### Supplementation of NMN combined with antibiotic treatment can alleviate liver inflammation

**Fig 6:**
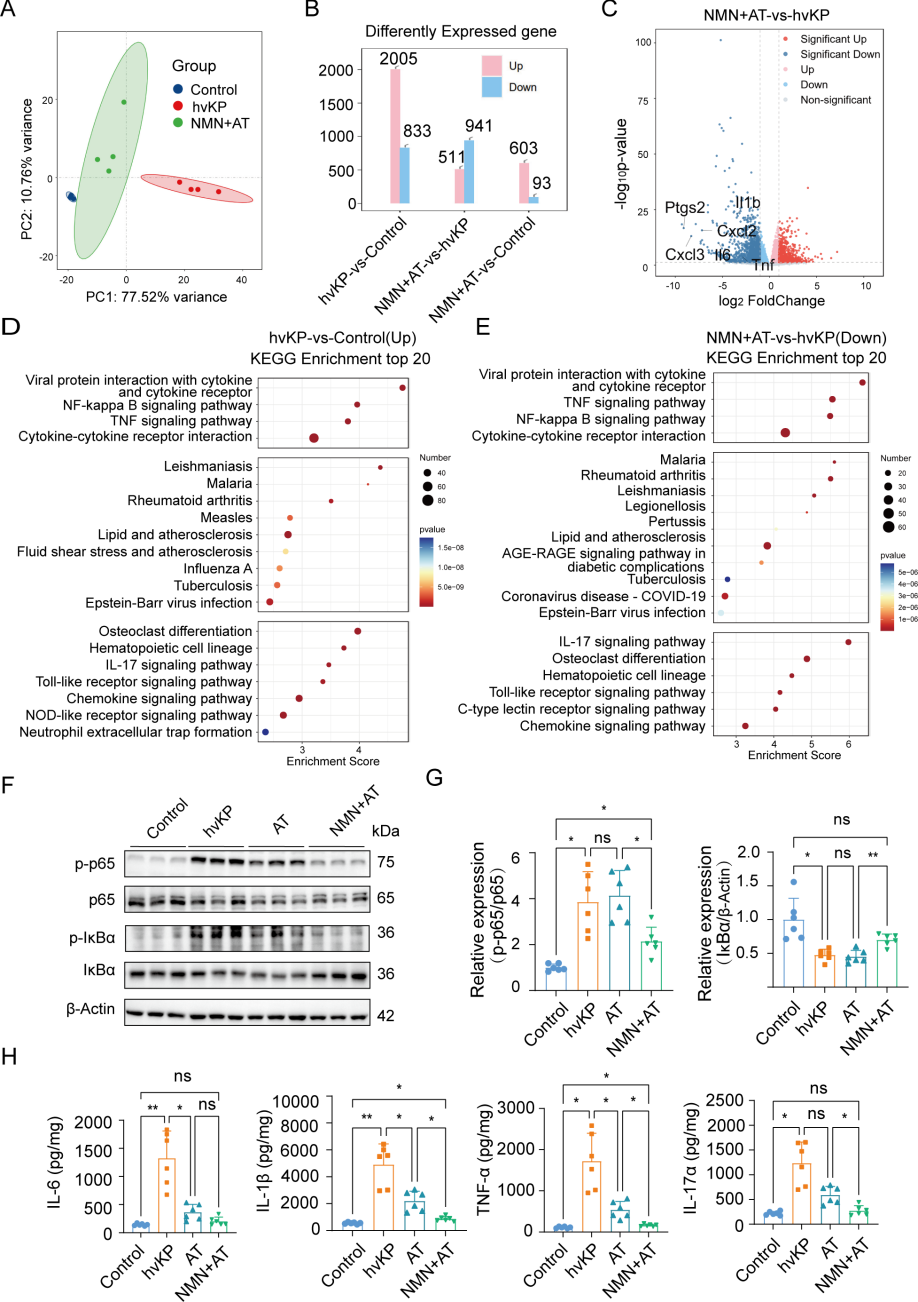
Supplementation of NMN combined with antibiotic treatment can alleviate liver inflammation. Genome-wide transcriptional sequencing results of the liver between control, hvKP infection, and NMN + antibiotic treatment (AT) groups (*n* = 4). (**A and B**) Principal component analysis and differentially expressed genes (DEGs) among three groups. (**C**) Volcano plot showing DEGs between hvKP infection group and the NMN + AT group. (**D**) Upregulated KEGG pathway enrichment after hvKP BSI. (**E**) Downregulated KEGG pathway enrichment after treatment with NMN + AT. (**F and G**) Western blot analysis of p-p65, p65, p- IκBα, and IκBα protein expression levels in liver among four groups (*n* = 6). (**H**) Levels of IL-6, IL-1β, TNF-α, and IL-17α in mouse liver among different groups (*n* = 6). Data are representative results from three independent experiments (mean  ±  SEM). *, *P* <  0.05; **, *P* <  0.01; ***, *P* <  0.001; ****, *P* < 0.0001; ns, not significant. NMN, β-nicotinamide mononucleotide; hvKP, hypervirulent *Klebsiella pneumoniae*; AT, antibiotic treatment after hvKP infection; Control, no treatment or infection group.

We compared the genome-wide transcriptional sequencing results of the liver between the control, hvKP infection, and NMN + antibiotic groups at 24 hpi. Principal component analysis showed that the transcriptome of the livers of mice treated with the combination of NMN and antibiotics was closer to that of the control group (Fig. 6A), and 2,005 significant differentially expressed genes (DEGs) were upregulated after hvKP BSI, while 941 DEGs were downregulated after NMN + antibiotics combination therapy (Fig. 6B). We compared gene changes in mice treated with the combination of NMN + antibiotics with those in the hvKP infection group and found that the mRNA levels of *Cxcl2*, *Cxcl3*, and *Ptgs2* related to neutrophil infiltration were significantly downregulated in top 20 genes, and the mRNA levels of *Il6*, *Il1b*, and *Tnf* were also downregulated (Fig. 6C). Next, we conducted gene ontology (GO) analysis to compare the intersections of increased function in hvKP infection and decreased function after treatment with NMN + antibiotics. It was found that the immune, inflammatory, and innate immune responses in the biological process subset of the mouse liver were significantly upregulated after hvKP BSI and decreased after treatment (Fig. S3A and B). Next, we conducted KEGG pathway analysis and compared the top 20 upregulated pathways after hvKP infection with those that were downregulated after treatment with NMN + antibiotics. The intersection of the two was used to identify pathways associated with hvKP liver abscesses. Based on the sequencing results, we found that the NF-κB and TNF signaling pathways, cytokine and cytokine receptor interactions, IL-17 signaling pathway, and Toll-like receptor signaling pathway were upregulated after infection and downregulated after treatment (Fig. 6D and E). The GSEA analysis also using the intersection method mentioned above revealed pathway enrichment of the NF-κB and TNF signaling pathways (Fig. S3C and D). Since antibiotic treatment significantly reduced liver CFUs, and NMN did not enhance bacterial clearance *in vivo*, we compared the activation status of the NF-κB pathway and the inflammatory cytokine levels in the liver tissue between antibiotic monotherapy and combination therapy with NMN + antibiotics to observe whether NMN supplementation could further reduce liver inflammatory response. The WB results showed that treatment with NMN + antibiotics can significantly reduce the ratio of phosphorylated p65 to p65 and increase IκBα levels in the liver (Fig. 6F and G). Enzyme-linked immunosorbent assay (ELISA) analysis of liver inflammatory factors showed that although antibiotic treatment could significantly reduce the levels of IL-6, IL-1β, and TNF-α in mouse liver, the combination of NMN could further reduce IL-1β, TNF-α, and IL-17α in mouse liver (Fig. 6H).

## DISCUSSION

HvKP BSI and liver abscesses are common diseases, but the immune mechanisms underlying the formation of liver abscesses are still not fully understood. The clearance of macrophages effectively reduced the infiltration of neutrophils into the liver and the formation of abscesses, indicating that the formation of hvKP liver abscess is not only related to bacterial load but also closely related to macrophages. The recruitment of neutrophils by liver macrophages is also crucial for liver abscess formation. Therefore, the immune response of hvKP-infected macrophages should receive more attention. To verify the hypothesis that liver abscess formation is related to excessive activation of the innate immune response (6), we constructed an antibiotic combination therapy model to evaluate the protective effects of different metabolites against hvKP liver abscess. Traditional liver pathology scoring is unsuitable for liver necrosis caused by bacterial liver abscesses. The pathological differences between the abscess and non-abscess areas were significant, and the final score was severely affected by the selected field of view. Through this model of combined antibiotics, we successfully quantified the protective effect as a visual relationship between “with abscess” and “without abscess.” At the same time, serum markers, such as ALT and LDH, also corresponded to the phenomenon observed by our naked eye, allowing visualization of the protective effect of NMN on liver abscesses. Our findings suggest that, in addition to bacterial load, the inflammatory response is likely also involved in the formation of liver abscesses. Moreover, liver abscess characteristics vary among different bacterial pathogens, requiring further investigation into animal models of liver abscess.

Our results indicated that NMN effectively suppresses NF-κB pathway activation and pro-inflammatory cytokine release in the hvKP BSI model. Both in *in vitro* and *in vivo* experiments, we did not find any promoting effect of NMN on bacterial clearance. We, therefore, propose that the liver protective effect of NMN on hvKP-induced liver injury is achieved by inhibiting excessive inflammatory response. Several studies have shown that common NAD^+^ supplements, including NMN, NR, and NAM, exhibit anti-inflammatory and anti-oxidant functions (9–11). The results of this study also support the inhibitory effect of NMN on the inflammatory response in the liver and BMDM. NR and NAM also blocked the formation of liver abscesses in a model of combined antibiotic treatment. This may be due to the use of high doses of NMN, NR, and NAM in our study, which may have significantly inhibited the inflammatory response *in vivo*. NMN, NR, and NAM are relatively safe, whereas other anti-inflammatory substances often cannot be administered at high doses. Therefore, NAD^+^ metabolism may be a promising research direction in hvKP infection.

Although previous studies have reported that NMN promotes the phagocytosis and bactericidal function of macrophages or neutrophils *in vitro* (12, 13), this study did not find any promotion of hvKP clearance by NMN supplementation *in vivo* or *in vitro*. Nonetheless, we found that FK866 can weaken the phagocytic functions of BMDM toward hvKP, and this weakening effect can be corrected by supplementation with NMN. The same effect was also observed in animal experiments, where NMN counteracted the increased bacterial load in the blood and liver caused by FK866. The above experiments demonstrate that the activity of NAMPT and NMN is necessary for mice to resist hvKP infection.

The limitations of this study are reflected in the following aspects: although NMN downregulates the activation of the NF-κB signaling pathway in the liver and BMDMs and reduces the levels of inflammatory factors, we still have not identified the key targets for the formation of hvKP liver abscesses. Our *in vivo* experiments were conducted following pre-supplementation with NMN. However, considering the different immune states *in vivo* during different stages of BSI, such as during the immune tolerance period of sepsis, NMN may not achieve good therapeutic effects, and its efficacy in clinical treatment should be studied further.

### Conclusion

NMN supplementation can improve the survival rate and reduce liver injury in mice with hvKP BSI by inhibiting excessive inflammatory response of macrophages and the liver.

## MATERIALS AND METHODS

### Mice and reagents

Eight-week-old wild-type male C57BL/6 mice were obtained from the Experimental Animal Center of the Anhui Province (Hefei, China). The mice were exposed to a 12 h light/dark cycle; humidity and temperature were controlled; and food and water were provided randomly for 5 days before starting the experiment. All animal experiments were approved by the Animal Experimentation Ethics Committee of the Anhui Medical University (approval no. LLSC20190253).

NMN (97%, cat. BD116593), FK866 (98%, cat. BD334954), and Meropenem (cat. BD137491) were acquired from Bidepharm, Shanghai, China; and NR (95%, cat. N885871), NAM (99%, cat. N814605), gentamycin sulfate (cat. G6064), and colistin sulfate salt (cat. C805491) were purchased from Macklin (Shanghai, China).

### Bacterial strains

*Klebsiella pneumoniae* (ATCC 43816, ATCC BAA-1705) was cultured overnight in Mueller-Hinton broth at 37°C. The cultures were diluted (1:100) with 5 mL fresh broth and grown until they reached the exponential phase. The number of bacterial CFUs based on 0.5 McFarland standard = 1.5  × 10^8^ CFU/mL.

### Models of hvKP BSI and treatment

Mice received intravenous injections with ATCC 43816 in 100 µL sterile phosphate-buffered saline (PBS) and were sacrificed at different time points post-infection. To quantify the bacterial burden in the blood, liver, and spleen, mice were sacrificed, and blood and organs were obtained, homogenized in PBS, and cultivated on Mueller-Hinton agar (MHA) plates. The survival status of mice was monitored every 6 h. Mice with near-death characteristics, such as mobility and breathing difficulties, were euthanized in advance to reduce pain. After intravenous injection of 4.0 × 10^7^ CFU of ATCC BAA-1705, mouse livers were obtained at 24 hpi. ATCC BAA-1705 did not cause liver abscesses at 24 hpi and served as the low-virulence control for cKP.

Clo-Lip (cat. 40337ES08) and control liposomes (cat. 40338ES08) were acquired from Yeasen, Shanghai, China. After macrophage depletion, mice were intravenously injected with 1.0 × 10^3^ CFU of ATCC 43816 to establish hvKP BSI.

For the survival experiments, NMN (185 mg/kg), NR (185 mg/kg), and NAM (150 mg/kg) were supplemented via intraperitoneal injection 1 h before infection, and FK866 (30 mg/kg) was administered 3 hpi via intraperitoneal injection. For the antibiotic combination therapy model, NMN (500 mg/kg), NR (500 mg/kg), and NAM (400 mg/kg) were administered via intraperitoneal injection 1 h before infection, and gentamicin (50 mg/kg), colistin (4 mg/kg), or meropenem (50 mg/kg) was administered at 4.5 hpi via intraperitoneal injection. This antibacterial drug administration method can eliminate bacteria from the blood of mice at 24 hpi while retaining the liver abscess phenotype of wild-type C57BL/6 mice. Doses of NMN, NR, and NAM were obtained from previous studies (10, 13, 14).

None of the animal experiments in this study required the addition of dimethyl sulfoxide to the drug carrier. Physiological saline was the injection carrier for NMN, NAM, and NR, while 20% sulfobutylether-β-cyclodextrin prepared in physiological saline was used for FK866. All drugs used in animal experiments were dissolved via intraperitoneal injection after vortex- and ultrasound-assisted dissolution. Control group mice received the corresponding carrier injections.

### Checkerboard assay

Prepare twofold serial dilutions of each antibiotic or NMN in a 96-well microplate. Culture the ATCC 43816 strain to exponential phase and adjust to 1.0 × 10⁶ CFU/mL in sterile broth. Add 50 µL bacterial suspension to each well (final volume: 100 µL/well; final concentration is 5 × 10⁵ CFU/mL). Seal and incubate at 37°C for 18–24 h, and measure OD600 to determine growth inhibition.

### Detection of biochemical parameters

Biochemical parameter detection kits were acquired from the Jian Cheng Bioengineering Institute (Nanjing, China). Next, we followed manufacturers’ instructions to determine alanine aminotransferase (ALT; cat. C009-2-1), aspartate aminotransferase (AST; cat. C010-2-1), and lactate dehydrogenase (LDH; cat. A020-2-2) levels.

### ELISA and NAD^+^ detection

Investigation of liver IL-6, IL-1β, TNF-α, and IL-17α was performed using IL-6 (cat. no. 431304), IL-1β (cat. no. 432604), TNF-α (cat. no. 430904), and IL-17α (cat. no. 432504) mouse ELISA kits, respectively, according to the manufacturer’s protocols (BioLegend). An NAD^+^ detection kit (cat. S0175) was acquired from Beyotime (Shanghai, China).

### Isolation of liver non-parenchymal cells

Liver non-parenchymal cells were separated and prepared as described previously (15). The mice were euthanized; a 27G needle was inserted into the inferior vena cava; and 10 mL of PBS was perfused. The liver was removed and cut into 1–2 mm^3^ pieces with blunt scissors. Samples were transferred to 50 mL Falcon tubes containing 10 mL preheated *ex vivo* digestive fluid (Roswell Park Memorial Institute medium [RPMI] 1640 [Gibco, USA] supplemented with 0.2 mg/mL collagenase (Sigma-Aldrich, USA), 5 units/mL of deoxyribonuclease I (Roche, Switzerland), and 10% fetal bovine serum (FBS; Gibco, USA). The sample was incubated at 37°C for 30 min and vortexed every 10 min at 2,000 rpm. The homogenized liver solution was passed through a 70 mm cell strainer into a clean 50 mL Falcon tube and washed with 10 mL of plain RPMI. After centrifugation at 50 × *g* for 3 min, the aqueous phase was recovered and transferred to a clean 50 mL Falcon tube and centrifuged at 400 × *g* for 5 min. Red blood cells were lysed, and after centrifugation, the pellet contained the target non-parenchymal cells, which were then incubated with an anti-mouse CD16/32 blocking antibody (BioLegend, USA, cat. 101320) for 15 min. Staining was performed with FITC anti-mouse CD45 (BioLegend, cat. 103108), APC/Cyanine7 anti-mouse CD11b (BioLegend, cat. 101226), and PE anti-mouse Ly6G (BioLegend, cat. 127608) for 30 min for on-machine analysis (FACS Celesta; BD Biosciences).

### Generation of bone marrow-derived macrophages (BMDMs)

After euthanizing the mice, bone marrow was extracted from the femur and tibia, and cells were cultured in Dulbecco's modified Eagle medium containing 10% FBS, 100 IU/mL penicillin, 100 µg/mL streptomycin, and 25 ng/mL macrophage colony-stimulating factor. The cultures were maintained at 37°C in 5% carbon dioxide, and on the sixth day, BMDMs were used for experiments.

### Real-time PCR

Total RNA was isolated using the TRIzol reagent (Invitrogen, USA) according to the manufacturer’s protocol. A cDNA synthesis kit (Takara, Japan) was used for cDNA synthesis according to the manufacturer’s instructions. Analysis was performed using SYBR Green (Takara) and a three-step real-time PCR system (Light Cycler 96; Roche). The primer sequences used in this study are listed in Table S1.

### Western blotting

Radioimmunoprecipitation assay lysate was added to the tissue and cell samples and lysed on ice for 30 min. Thereafter, samples were centrifuged at 12,000 × *g* for 10 min, and SDS-PAGE protein loading buffer was added, followed by heating to completely denature the protein. Electrophoresis, membrane transfer, and sealing of samples were performed. For total protein, membranes were incubated overnight with the following antibodies: p65 (Proteintech, Wuhan, China, cat. 10745-1-AP), p-p65 (Proteintech, cat. 82335-1-RR), IκBα (Proteintech, cat. 10268-1-AP), p-IκBα (Abcam, USA, cat. ab92700), and β-actin (Proteintech, cat. 66009-1-Ig). The secondary antibody was incubated at room temperature for 1 h, and the ECL western blotting detection system was used to identify proteins.

### Phagocytosis and bacterial killing assay

Phagocytosis and bacterial killing assays were performed as previously described (16). ATCC-43816 was added according to the multiplicity of infection (MOI) of 100, incubated for 1 h, and then replaced with a medium containing 100 µg/mL of gentamicin for 30 min. After washing thrice with PBS, the cells were lysed with 0.3% (vol/vol) Triton X-100 for 5 min. The cell lysate was continuously diluted with PBS and seeded onto MHA plates to count the CFU. For phagocytic experiments, GFP-labeled ATCC-43816 was incubated for 1 h (MOI = 100), and phagocytic activity was detected using flow cytometry.

### Histopathological examination and immunohistochemistry (IHC)

Liver tissue was fixed in 4% paraformaldehyde. Paraffin-embedded liver tissues were cut into 5-μm-thick sections and processed for hematoxylin and eosin analysis. After dewaxing, hydration, and antigen retrieval, sections were incubated with anti-F4/80 (Proteintech, cat. 28463-1-AP) and anti-MPO (Proteintech, cat. 22225-1-AP) at 4°C overnight. A color reaction was performed using an HRP-linked polymer detection system and counterstained with hematoxylin before dehydration in graded alcohol.

### Immunofluorescence assay

Cells were fixed with 4% paraformaldehyde for 15 min and permeabilized with 0.5% Triton X-100 at room temperature for 30 min. Fixed and permeabilized cells were blocked with 5% bovine serum albumin PBS for 1 h, then incubated with anti-p65 primary antibody at 4°C overnight, followed by washing with PBST three times and incubation with secondary antibody for 1 h. In addition, cells were stained with Hoechst 33258 (Solarbio, Beijing, China, cat. C0021) to determine the nuclear boundaries. Images were captured using a Carl Zeiss LSM-800 confocal microscope (Germany).

### RNA-seq

Total RNA was extracted using TRIzol reagent according to the manufacturer’s protocol. Libraries were constructed using the VAHTS Universal V6 RNA-seq Library Prep Kit according to the manufacturer’s instructions and sequenced on Illumina Novaseq 6000 platform, and 150 bp paired-end reads were generated. Raw reads in fastq format were first processed using fastp1, and low-quality reads were removed to obtain clean reads, which were mapped to the reference genome using HISAT2. The FPKM of each gene was calculated, and the read counts of each gene were obtained using HTSeq-count. Principal component analysis was performed using R (v 3.2.0) to evaluate biological duplication of samples, and differential expression analysis was performed using DESeq. A *Q* value < 0.05, fold change > 2, or fold change < 0.5 was set as the threshold for significantly differentially expressed genes (DEGs). Hierarchical cluster analysis of DEGs was performed using R (v 3.2.0) to demonstrate the expression pattern of genes in different groups and samples. Based on the hypergeometric distribution, GO, KEGG pathway enrichment analysis of DEGs was performed to screen significantly enriched terms (17, 18). R (v 3.2.0) was used to draw the column and bubble diagrams of significantly enriched terms. Gene set enrichment analysis (GSEA) was performed using GSEA software (19).

### Statistical analysis

Data were obtained from at least three independent experiments. The mean ± standard errors of the mean (SEM) was used to express the results. Unpaired Student’s *t* tests were used for two-group comparisons, whereas one-way or two-way analysis of variance was used for multiple-group analyses. *P* values less than 0.05 were considered statistically significant. All graphs were generated using Adobe Illustrator 2021 or GraphPad Prism version 9.

## Data Availability

All RNA-seq data have been submitted to the NCBI Sequence Read Archive (SRA) under BioProject accession no. PRJNA1279787.

## SUPPLEMENTAL MATERIAL

Supplemental Material msphere.00361-25-s0001.pdf Figures S1 to S3; Table S1.

ASM does not own the copyrights to Supplemental Material that may be linked to, or accessed through, an article. The authors have granted ASM a non-exclusive, world-wide license to publish the Supplemental Material files. Please contact the corresponding author directly for reuse.

## References

[1] Lamy B, Sundqvist M, Idelevich EA. 2020. Bloodstream infections - standard and progress in pathogen diagnostics. Clin Microbiol Infect 26:142–150. doi:10.1016/j.cmi.2019.11.017 31760113

[2] Russo TA, Marr CM. 2019. Hypervirulent Klebsiella pneumoniae. Clin Microbiol Rev 32:e00001-19. doi:10.1128/CMR.00001-19 31092506 PMC6589860

[3] Huang X, Li X, An H, Wang J, Ding M, Wang L, Li L, Ji Q, Qu F, Wang H, Xu Y, Lu X, He Y, Zhang JR. 2022. Capsule type defines the capability of Klebsiella pneumoniae in evading Kupffer cell capture in the liver. PLoS Pathog 18:e1010693. doi:10.1371/journal.ppat.1010693 35914009 PMC9342791

[4] Hoh CH, Tan YH, Gan YH. 2019. Protective role of kupffer cells and macrophages in klebsiella pneumoniae-induced liver abscess disease. Infect Immun 87:e00369-19. doi:10.1128/IAI.00369-19 31285251 PMC6704610

[5] Wanford JJ, Hames RG, Carreno D, Jasiunaite Z, Chung WY, Arena F, Di Pilato V, Straatman K, West K, Farzand R, Pizza M, Martinez-Pomares L, Andrew PW, Moxon ER, Dennison AR, Rossolini GM, Oggioni MR. 2021. Interaction of Klebsiella pneumoniae with tissue macrophages in a mouse infection model and ex-vivo pig organ perfusions: an exploratory investigation. Lancet Microbe 2:e695–e703. doi:10.1016/S2666-5247(21)00195-6 34901898 PMC8641047

[6] Hullahalli K, Dailey KG, Hasegawa Y, Torres E, Suzuki M, Zhang H, Threadgill DW, Navarro VM, Waldor MK. 2023. Genetic and immune determinants of E. coli liver abscess formation. Proc Natl Acad Sci USA 120:e2310053120. doi:10.1073/pnas.2310053120 38096412 PMC10743367

[7] Yoshino J, Baur JA, Imai S. 2018. NAD+ Intermediates: the biology and therapeutic potential of NMN and NR. Cell Metab 27:513–528. doi:10.1016/j.cmet.2017.11.002 29249689 PMC5842119

[8] Chini CCS, Cordeiro HS, Tran NLK, Chini EN. 2024. NAD metabolism: role in senescence regulation and aging. Aging Cell 23:e13920. doi:10.1111/acel.13920 37424179 PMC10776128

[9] Liu LW, Xie Y, Li GQ, Zhang T, Sui YH, Zhao ZJ, Zhang YY, Yang WB, Geng XL, Xue DB, Chen H, Wang YW, Lu TQ, Shang LR, Li ZB, Li L, Sun B. 2023. Gut microbiota-derived nicotinamide mononucleotide alleviates acute pancreatitis by activating pancreatic SIRT3 signalling. Br J Pharmacol 180:647–666. doi:10.1111/bph.15980 36321732

[10] Hong G, Zheng D, Zhang L, Ni R, Wang G, Fan GC, Lu Z, Peng T. 2018. Administration of nicotinamide riboside prevents oxidative stress and organ injury in sepsis. Free Radic Biol Med 123:125–137. doi:10.1016/j.freeradbiomed.2018.05.073 29803807 PMC6236680

[11] Curran CS, Dougherty EJ, Cui X, Li Y, Jeakle M, Gamble T, Demirkale CY, Torabi-Parizi P. 2023. Nicotinamide antagonizes lipopolysaccharide-induced hypoxic cell signals in human macrophages. J Immunol 211:261–273. doi:10.4049/jimmunol.2200552 37314413 PMC10315438

[12] Cros C, Margier M, Cannelle H, Charmetant J, Hulo N, Laganier L, Grozio A, Canault M. 2022. Nicotinamide mononucleotide administration triggers macrophages reprogramming and alleviates inflammation during sepsis induced by experimental peritonitis. Front Mol Biosci 9:895028. doi:10.3389/fmolb.2022.895028 35832733 PMC9271973

[13] Cao T, Ni R, Ding W, Ji X, Fan GC, Zhang Z, Peng T. 2023. Nicotinamide mononucleotide as a therapeutic agent to alleviate multi-organ failure in sepsis. J Transl Med 21:883. doi:10.1186/s12967-023-04767-3 38057866 PMC10699070

[14] Yuan H, Wan J, Li L, Ge P, Li H, Zhang L. 2012. Therapeutic benefits of the group B3 vitamin nicotinamide in mice with lethal endotoxemia and polymicrobial sepsis. Pharmacol Res 65:328–337. doi:10.1016/j.phrs.2011.11.014 22154801

[15] Andreata F, Blériot C, Di Lucia P, De Simone G, Fumagalli V, Ficht X, Beccaria CG, Kuka M, Ginhoux F, Iannacone M. 2021. Isolation of mouse Kupffer cells for phenotypic and functional studies. STAR Protoc 2:100831. doi:10.1016/j.xpro.2021.100831 34585164 PMC8450292

[16] Wu T, Li H, Su C, Xu F, Yang G, Sun K, Xu M, Lv N, Meng B, Liu Y, Hu L, Liu Y, Gao Y, Wang H, Lan Y, Xu D, Li J. 2020. Microbiota-derived short-chain fatty acids promote LAMTOR2-mediated immune responses in macrophages. mSystems 5:e00587-20. doi:10.1128/mSystems.00587-20 PMC7646525 33144310

[17] The Gene Ontology Consortium. 2019. The gene ontology resource: 20 years and still GOing strong. Nucleic Acids Res 47:D330–D338. doi:10.1093/nar/gky1055 30395331 PMC6323945

[18] Kanehisa M, Araki M, Goto S, Hattori M, Hirakawa M, Itoh M, Katayama T, Kawashima S, Okuda S, Tokimatsu T, Yamanishi Y. 2008. KEGG for linking genomes to life and the environment. Nucleic Acids Res 36:D480–4. doi:10.1093/nar/gkm882 18077471 PMC2238879

[19] Subramanian A, Tamayo P, Mootha VK, Mukherjee S, Ebert BL, Gillette MA, Paulovich A, Pomeroy SL, Golub TR, Lander ES, Mesirov JP. 2005. Gene set enrichment analysis: a knowledge-based approach for interpreting genome-wide expression profiles. Proc Natl Acad Sci USA 102:15545–15550. doi:10.1073/pnas.0506580102 16199517 PMC1239896

