# Supplementary material for: β-Nicotinamide mononucleotide protects against hypervirulent Klebsiella pneumoniae bloodstream infection and liver injury
Source: mSphere. 2025 Jul 31;10(8):e00361-25. doi: 10.1128/msphere.00361-25 (PMC12379607; doi:10.1128/msphere.00361-25)
Supplement: Supplemental Material — Figures S1 to S3; Table S1. [file msphere.00361-25-s0001.pdf]

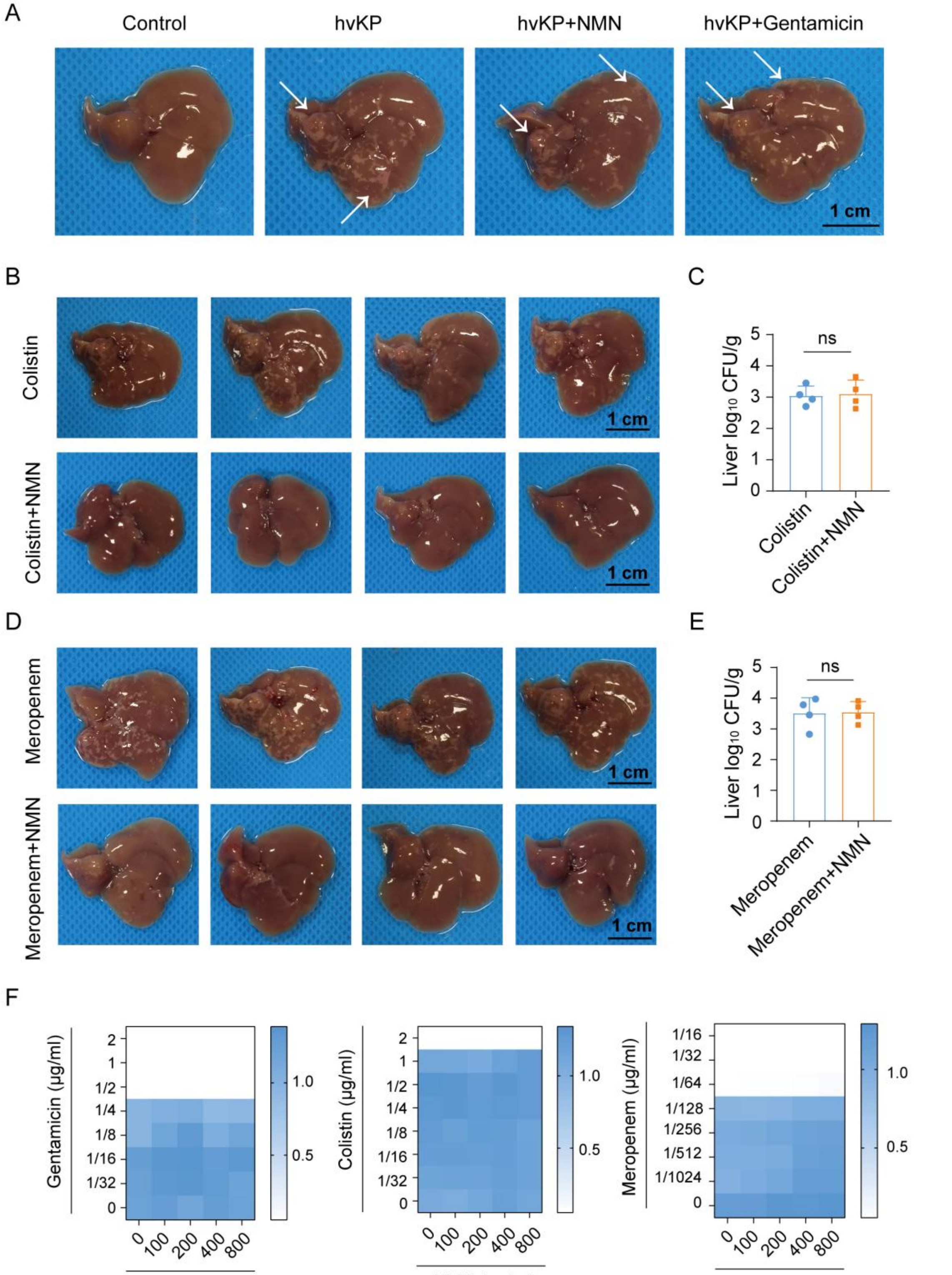

Figure S1. Supplementation of NMN combined with antibiotic treatment to alleviate liver injury and abscess caused by hvKP. (A) Representative images of mouse liver

in different groups. (B-C) Images of mouse liver and bacterial load after treatment with colistin alone or NMN supplementation combined with colistin therapy (n=4). (D-E) Images of mouse liver and bacterial load after treatment with meropenem alone or NMN supplementation combined with meropenem therapy (n=4). (F) Checkerboard assay results of different antibiotics combined with NMN treatment, with a heatmap of OD600 values. Data are representative results from three independent experiments. *, $p < 0.05$; **, $p < 0.01$; ***, $p < 0.001$; ****, $p < 0.0001$; ns, not significant. Control, no treatment or infection group; hvKP, hypervirulent *Klebsiella pneumoniae*; NMN, β-nicotinamide mononucleotide; CFU, colony-forming units.

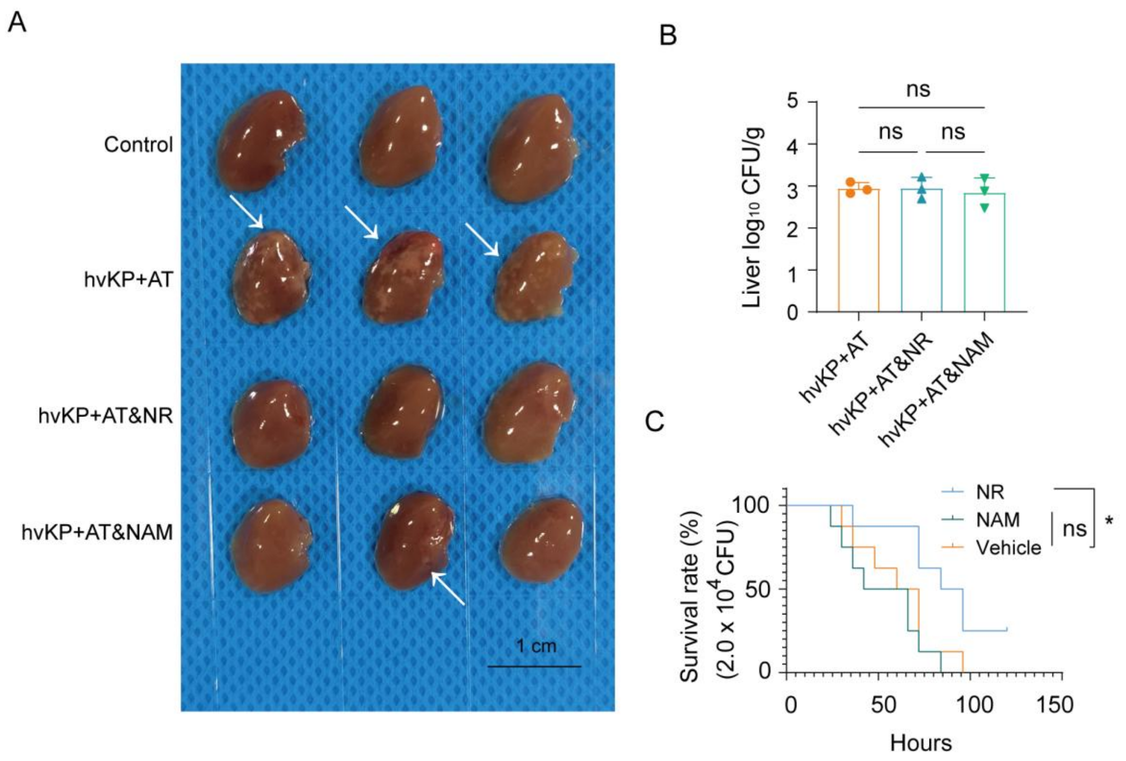

Figure S2. Supplementation of NR or NAM combined with antibiotic treatment to alleviate liver injury and abscess caused by hvKP. (A) Mouse liver right upper lobes in different groups (n=3). (B) Bacterial load of mouse liver in different groups (n=3). (C) Survival rates of NR or NAM supplemented group and vehicle group infected with 2.0×10^4 CFU of ATCC 43816 (n=8). Data are representative results from three independent experiments. *, $p < 0.05$; **, $p < 0.01$; ***, $p < 0.001$; ****, $p < 0.0001$; ns, not significant. AT, antibiotic treatment after hvKP infection; NR, nicotinamide nucleoside; NAM, nicotinamide; CFU, colony-forming units. Control, no treatment or infection group; Vehicle, vehicle treatment and infection group.

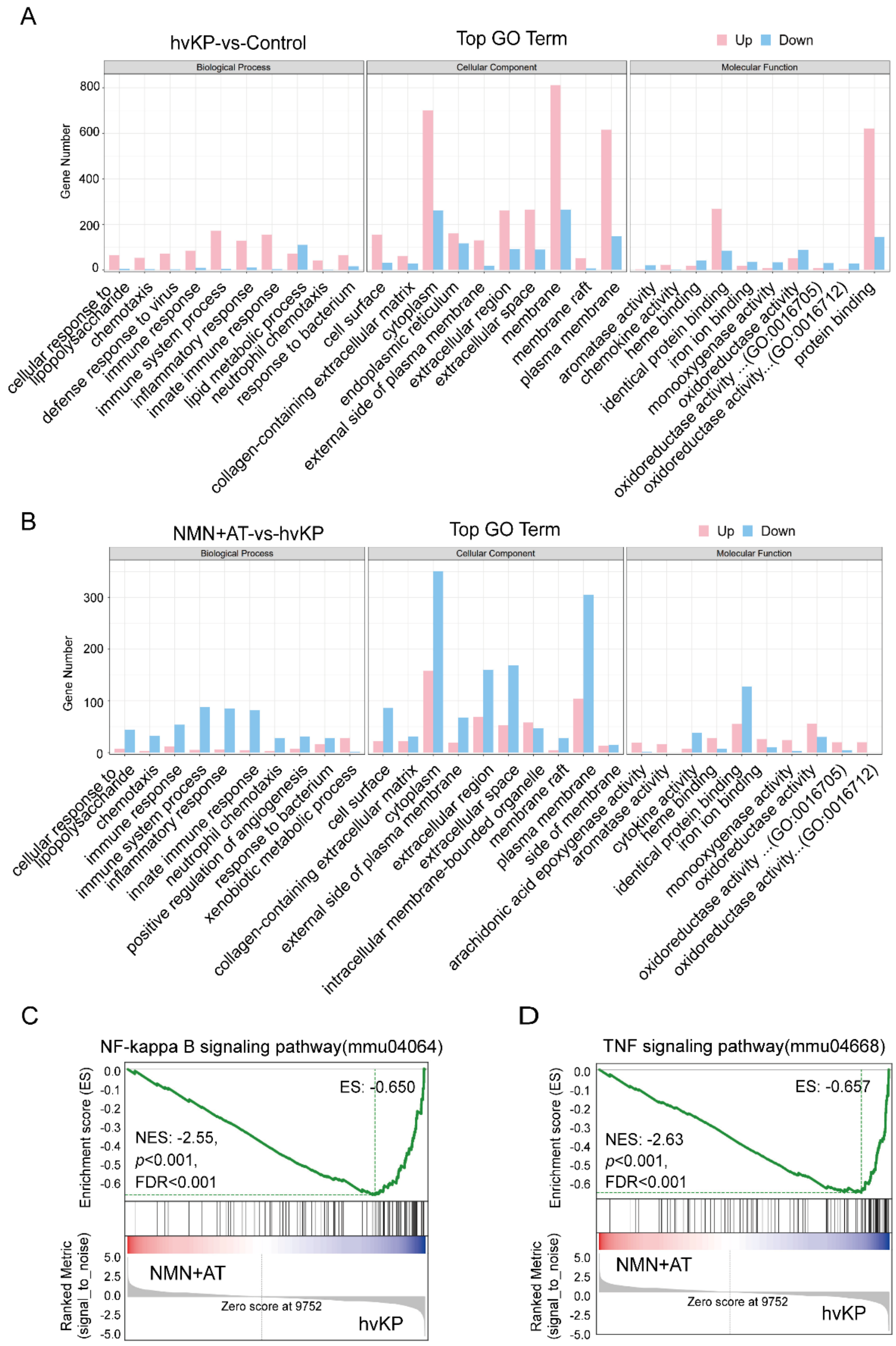

Figure S3. GO analysis and GSEA analysis. (A) TOP GO terms after hvKP BSI. (B)

TOP GO terms after treatment with NMN + antibiotics. (C-D) GSEA analysis of NF-κB signaling pathway and TNF signaling pathway after treatment with NMN + antibiotics. hvKP, hypervirulent *Klebsiella pneumoniae*; NMN, β-nicotinamide mononucleotide; AT, antibiotic treatment after hvKP infection; Control, no treatment or infection group.

Table.S1 Primers for Real-Time PCR

Genes	Forward (5′–3′)	Reverse (5′–3′)
Actb	GTGACGTTGACATCCGTAAAGA	GCCGGACTCATCGTACTCC
Gapdh	AGGTCGGTGTGAACGGATTTG	GGGGTCGTTGATGGCAACA
Tdo2	TGGCAATTACTTGCAGTTGGA	GTGCTCGTCATGGATTTTGTTC
Qprt	CATCCTTGGTTACCGGGTCG	GCCAGGGTGTTAAGAGCCA
Naprt	TGCTCACCGACCTCTATCAGG	GCGAAGGAGCCTCCGAAAG
Nampt	GCAGAAGCCGAGTTCAACATC	TTTTCACGGCATTCAAAGTAGGA
Nmnat1	TGGCTCTTTTAACCCCATCAC	TCTTCTTGTACGCATCACCGA
Nmnat2	ATGACCGAGACCACAAAGACC	ATCCCGCCAATCACAATAAATCT
Nmnat3	ATCACGAATATGCACCTGCG	ATTGACGGGTGAGATGATGCC
Il6	CTGCAAGAGACTTCCATCCAG	AGTGGTATAGACAGGTCTGTTGG
Il1b	GAAATGCCACCTTTTGACAGTG	TGGATGCTCTCATCAGGACAG
Tnf	CAGGCGGTGCCTATGTCTC	CGATCACCCCGAAGTTCAGTAG
Cxcl2	CCAACCACCAGGCTACAGG	GCGTCACACTCAAGCTCTG
